# EZH2 promotes endometriosis progression through estrogen receptor and TNFα expression

**DOI:** 10.3389/fendo.2025.1574938

**Published:** 2025-06-24

**Authors:** Xiaohan Liu, Liqin Cheng, Liuxuan Huang, Mingyue Li, Qingjun Shen, Donghan Li, Kailing Dai, Yanxia Fu, Min Li, Paul Yao, Liqin Zeng

**Affiliations:** ^1^ Department of Gynecology, The Eighth Affiliated Hospital, Sun Yat-Sen University, Shenzhen, China; ^2^ Institute of Burns, Tongren Hospital of Wuhan University (Wuhan Third Hospital), Wuhan, China

**Keywords:** endometriosis, estrogen receptor, EZH2, inflammation, TNFα

## Abstract

Endometriosis is a chronic inflammatory gynecological condition marked by the presence of tissue similar to the endometrium grows outside the uterus, often leading pelvic pain and infertility. This study explores how enhancer of zeste homolog 2 (EZH2) influences endometriosis, particularly through its interaction with estrogen receptors (ERs). We found that EZH2 reduces ERα expression, allowing ERβ to bind to the tumor necrosis factor α (TNFα) promoter and increase TNFα levels, fueling inflammation. In mice, the EZH2 inhibitor GSK343 reduced TNFα levels and endometriosis progression, similar to gene knockdown of ERβ or EZH2. In human samples, endometriotic tissue showed higher levels of EZH2 and ERβ and lower levels of ERα than in controls. Thus, EZH2 promotes TNFα-driven inflammation, contributing to endometriosis. Targeting EZH2, as with GSK343, could be a promising therapeutic strategy for endometriosis treatment.

## Introduction

Endometriosis (EMS) is a long-term gynecological disorder marked by inflammation and the growth of estrogen-driven lesions similar to endometrial tissue found outside the uterus. This disease often results in pelvic pain and infertility (1, 2). While the precise cause of EMS remains unclear, contributing factors are thought to include retrograde menstruation, immune system dysfunction, hormonal influences, and environmental exposures (3–6). Elevated levels of estrogen receptor β (ERβ) have been observed in EMS lesions, though its role and effects in EMS progression are not fully understood (7, 8).

Enhancer of zeste homolog 2 (EZH2) is an important component of polycomb repressive complex and acts primarily as a histone methyltransferase, facilitating the methylation of histone H3 at lysine 27, known as H3K27me3. This methylation leads to chromatin condensation and transcriptional repression of target genes (9, 10). EZH2 is associated with endometriosis development, as endometriotic lesions exhibit significantly higher levels of EZH2 (11, 12), although the possible mechanism is still not well understood. Here, our goal is to examine the role of EZH2 in EMS development by examining its influence on estrogen receptors and the regulation of tumor necrosis factor α (TNFα) expression (13).

EZH2 has been implicated in the regulation of several pro-inflammatory genes through H3K27me3 modification, often leading to gene silencing (14). Several studies suggest that inhibiting EZH2 can reduce tissue inflammation (15, 16). Our preliminary data indicate that EZH2 expression enhances TNFα levels, suggesting that EZH2 may promote inflammatory pathways through mechanisms not yet fully understood, possibly indirectly affecting TNFα production. Estrogen receptors (ERs), including ERα and ERβ, also contribute to inflammatory responses either through the genomic pathway-directly binding to estrogen response element (ERE) on DNA to regulate gene transcription (17, 18) or through non-genomic pathways, such as NFκB and MAPK, which indirectly influence inflammation-related gene expression (19, 20). We hypothesize that EZH2 regulates inflammation and EMS development via interactions with ERs, making EZH2 a potential therapeutic target for managing EMS-associated inflammation and symptoms.

In this research, we aim to investigate the mechanisms of EZH2-mediated inflammation and EMS progression through ERs and TNFα expression. Human endometrial stromal cells (HESC) and epithelial cells (HEEC) were used *in vitro* to investigate EZH2-mediated ERα suppression via H3K27me3 modification and the subsequent upregulation of TNFα through competitive binding of ERα and ERβ to the ERE on the TNFα promoter. Additionally, a mixture of HEEC and HESC cells treated by latent membrane protein 1 (LMP1) was transplanted to create an endometriotic mouse model (21) to examine the effects of the EZH2 inhibitor GSK343 (22) on inflammation and EMS development. Finally, tissue samples from 50 human subjects, including both EMS patients and controls, were examined for the gene expression of EZH2, estrogen receptors, TNFα, and markers of oxidative stress to corroborate the findings from *in vitro* and animal studies.

## Materials and methods

The details for this section are available from supplementary material Data S1, and the sequences of primers used in this article are available from **Supplementary Table S1**.

### Reagents and materials

Both HEEC and HESC cells were used in this study at passage 3. In certain experiments, they were conditionally immortalized with a human telomerase reverse transcriptase (hTERT) lentiviral vector to extend lifespan (23). 17β-estradiol (E2, #E2758) and the EZH2 inhibitor GSK343 (#SML0766, in 0.1% DMSO) were purchased from Sigma, China (24). Antibodies for ERα (sc-8005), c-Rel (sc-6955), Ki-67 (sc-101861), NF1 (sc-74444) and TNFα (sc-52746) were sourced from Santa Cruz Biotechnology. Antibody for EZH2 (ab186006) was purchased from Abcam.

### Construction of reporter plasmids

Human ERα and TNFα reporter plasmids were constructed by amplifying the promoter regions (2 kb upstream of TSS + first exon) from human genomic DNA using Ensembl IDs ESR1–201 ENST00000206249.8 (ERα) and TNF-201 ENST00000376122.3 (TNFα). The promoter fragments were then inserted into pGL3-basic plasmid through KpnI and XhoI sites with below primers: ERα forward: 5’-gcgc-ggtacc- cac aca ctc tct ctg cct agt -3’ (Kpn1) and ERα reverse: 5’- gtac- ctcgag- ctg tag aat gcc ggc ggg ccg -3’ (Xho1); TNFα forward: 5’-gcgc-ggtacc-gca ctc gat gta cca cgg ggc -3’ (Kpn1) and TNFα reverse: 5’- gtac- ctcgag- ctc ttc cct ctg ggg gcc gat -3’ (Xho1).

### Methods

mRNA levels were determined by quantitative PCR using primers indicated in **Supplementary Table S1**. Protein expression was measured by western blotting and evaluated through immunostaining or immunohistochemistry (IHC). Promoter reporter activity was determined by luciferase reporter assay, and the association of factors with gene promoters was assessed by chromatin immunoprecipitation (ChIP). Oxidative stress was evaluated by ROS production using CM-H2DCFDA-based fluorescence assay (25), alongside analysis of the GSH/GSSG ratio, 3-nitrotyrosine formation, γH2AX formation, and 8-oxo-dG formation. Cytokines IL1β, IL6, and TNFα were quantified with ELISA kits from R&D Systems, as was PGE2 (26).

### Immunostaining

Cells on coverslips were fixed, permeabilized, and incubated with primary antibodies (Ki-67 or 8-oxo-dG) followed by FITC/Texas Red-labeled secondary antibodies. Slides were stained with DAPI (#D9542, Sigma) and images analyzed with ImageJ.

### [^3^H]-thymidine incorporation for DNA synthesis

Cells were incubated with [3H]-thymidine in serum-free media, washed, and DNA precipitated with trichloroacetic acid. DNA synthesis was measured as counts per minute (CPM) in a scintillation counter (27).

### Soft agar colony formation assay

Cells were seeded in 0.3% agarose over a 0.5% agarose layer in DMEM with 5% FBS. After 30 days, colonies were scored if they reached at least 50mm in diameter, with experiments performed in quadruplicate.

### *In vivo* mouse experiments

#### Animal care and procedures

All animal protocols were approved by the Institutional Animal Care and Use Committee of the Affiliated No. 8 Hospital of Sun Yat-Sen University. Four-week-old female NSG mice (sourced from Jackson Lab) underwent ovariectomy, followed by subcutaneous implantation of a 17β-estradiol (E2) pellet.

#### Transplantation and treatment in mouse model

Both HESC and HEEC were pretreated by LMP1 adenovirus for 2 days; then cultured continuously to isolate single colony until passage 6 (21), then cells were subsequently infected by either empty (EMP), shEZH2, or shERβ lentivirus for transplantation. On the transplantation day, cells were trypsinized, and a mixture of 2 × 10⁶ HESC and HEEC cells was resuspended in a 1:1 Matrigel (BD Biosciences, Beijing) mix (total volume 150 µl), then maintained on ice. The cell mixture was injected intraperitoneally, avoiding the peritoneal layer and organs (28). Mice were then randomly separated into below four groups (1): EMP/VEH, empty lentivirus control cells with vehicle injection (2); shERβ/VEH, shERβ-treated cells with vehicle injection (3); shEZH2/VEH, shEZH2-treated cells with vehicle injection; and (4) EMP/GSK343, empty lentivirus control cells with 10 mg/kg body weight of GSK343 injection every two days for four weeks.

#### Endometriosis lesion assessment

At four weeks post-transplantation, mice were euthanized by CO₂ asphyxiation. Blood was withdrawn and the serum and peripheral blood mononuclear cells (PBMC) were prepared. Endometriosis lesions in the abdominal cavity were identified through gross visual examination, and their numbers and sizes were documented. Lesions were classified as either single or multiple (2 or 3) nodules (28, 29). PBMC was used to assess oxidative stress, serum was employed determine GSH/GSSG ratio and pro-inflammatory cytokine levels. Gene expression of lesion samples was determined (30). Remaining peritoneal tissue was fixed and processed for immunohistochemical analysis (28).

### Immunohistochemistry

Cryostat-cut sections (10 µm) of endometriotic lesions were fixed, permeabilized and incubated with primary antibodies (40 µg/ml) for ERα, ERβ, EZH2, Ki67, TNFα, or 8-oxo-dG for 2 hours, then incubated with FITC-labeled secondary antibodies (for animal tissues) or HRP-labeled secondary antibodies (for human tissues) for one hour, followed by DAPI nuclear staining for fluorescence analysis or diaminobenzidine (DAB) for chromogenic visualization. Images were captured, and protein expression was quantified using ImageJ software (29, 31).

### Human subjects study

The study protocol was reviewed and approved by the Institutional Ethical Committee of The Eighth Affiliated Hospital of Sun Yat-Sen University, and informed written consent was obtained from each participant.

#### Inclusion and exclusion criteria

Eligible participants were women aged 18–49 years who had not reached menopause, with no use of hormonal medications in the 3 months prior to enrollment. Diagnosis was confirmed by two pathologists according to the following criteria: microscopic examination showed endometriotic lesions with endometrial glands, stroma, hemorrhagic areas, and hemosiderin. Exclusion criteria included postmenopausal women, use of hormonal medications in the 3 months prior to enrollment, and postoperative pathology revealing malignancy or non-endometriotic cysts.

#### Study groups

A total of 50 subjects were enrolled in both endometriotic and control groups. For the endometriotic group, diagnosis was confirmed through surgical and histological verification. Participants in the control group, who underwent surgery for benign gynecologic conditions, met the following criteria (1): no visible endometriosis (2); similar age and reproductive status to the endometriotic group (3); exclusion of other gynecological conditions (4); similar lifestyle factors (5); no use of hormone-altering medications; and (6) no chronic pain symptoms. These criteria ensure that differences between the control and experimental groups are primarily due to endometriosis, enhancing the scientific validity and interpretability of the results (32). Clinical and demographic data for participants are presented in **Table 1**.

**Table 1:** Clinical characteristics of women with endometriosis and control group.

Patient characteristics	Control group (n=50)	Endometriosis group (n=50)	*P* value (t or χ^2^ test)
Age (years)	36.8 ± 6.1	34.6 ± 7.8	0.483
BMI (kg/m^2^)	23.5 ± 2.4	21.8 ± 1.9	0.071
Menstrual average cycle (days)	27.4 ± 3.9	26.8 ± 4.3	0.874
Menstrual duration (days)	6.3 ± 1.5	6.4 ± 1.5	0.912
Menstrual cycle phase			
Proliferative phase	29	26	0.056
Secretory phase	21	19	0.078
Unknown phase	0	5	<0.001*
Disease localization			
Peritoneum	N/A	2 (4%)	N/A
Fallopian tube	N/A	7 (14%)	N/A
Sacrouterine lig	N/A	41 (82%)	N/A
Symptoms			
Pain	N/A	35 (70%)	N/A
Infertility	N/A	25 (30%)	N/A
ASRM stage			
Stage 1/2	N/A	0 (0%)	N/A
Stage 3	N/A	20 (40%)	N/A
Stage 4	N/A	30 (60%)	N/A

Data are presented as mean ± standard deviation. *significant difference at *P <*0.05. ASRM, American Society for Reproductive Medicine; BMI, body mass index; N/A, not applicable.

#### Tissue collection and analysis

Collected endometrial tissues were analyzed for mRNA levels by qPCR and immunohistochemical staining for ERα, ERβ, EZH2, TNFα, and 8-oxo-dG.

## Results

### EZH2 suppresses ERα expression via H3K27me3 modification and Sp3 dissociation from the ERα promoter

**Figure 1:**
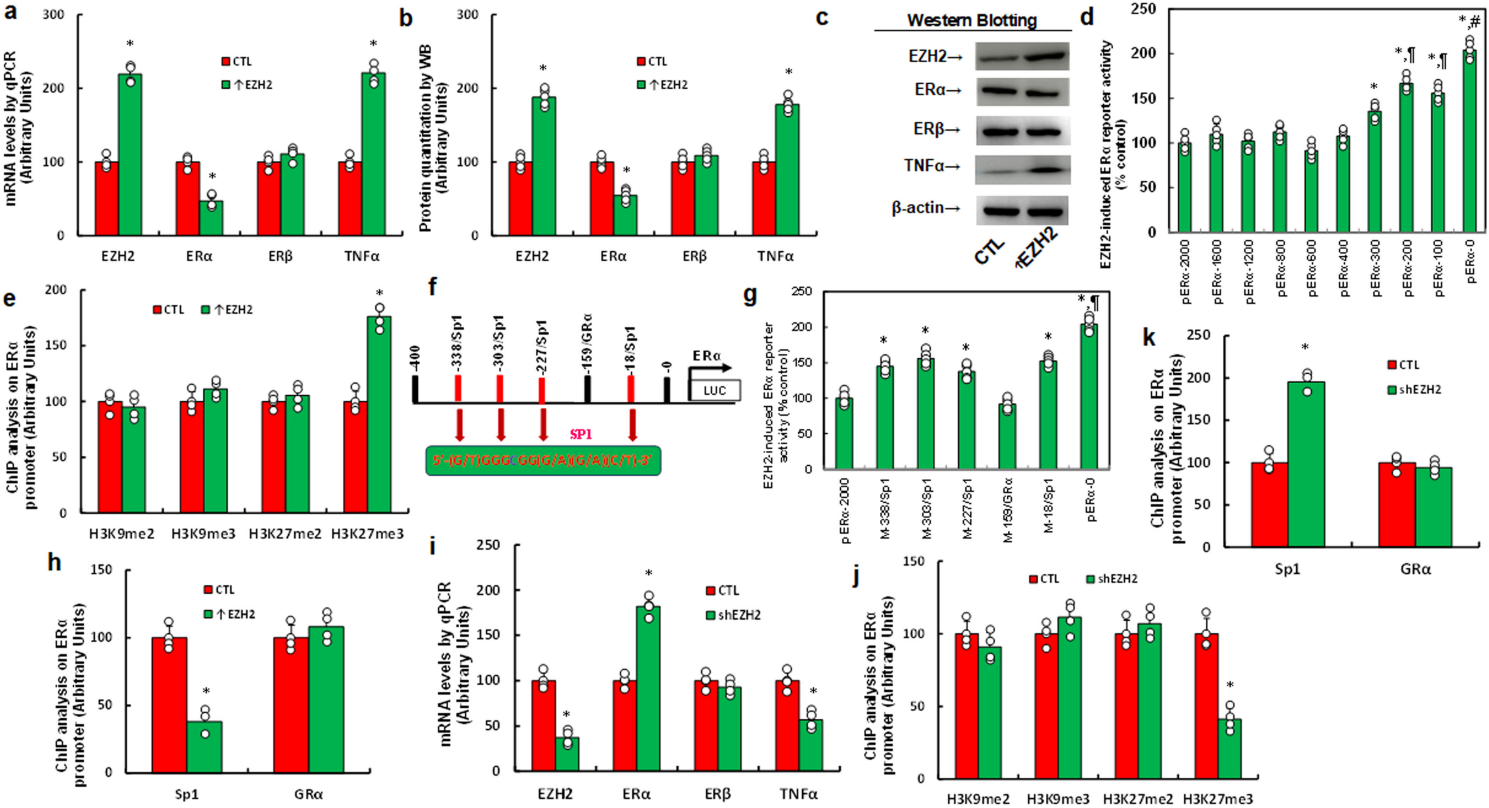
EZH2 suppresses ERα expression via H3K27me3 modification and subsequent dissociation of Sp3 from the ERα promoter. **(a-c)** Immortalized human HESC cells were infected with either control (CTL) or EZH2 expression lentivirus (↑EZH2) for 48 hours before undergoing biological assays. **(a)** mRNA levels were measured using qPCR, n=4. **(b)** Protein quantification was performed via western blotting, n=5. **(c)** Representative blots for **(b)**. *P<0.05 compared to the CTL group. **(d)** Immortalized HESC cells were transfected with ERα full-length or deletion reporter constructs for a luciferase activity assay, n=5. *P<0.05 vs pERα-2000 group; ¶, P<0.05 vs pERα-300 group; #, P<0.05 vs pERα-200 group. **(e)** ChIP analysis of the ERα promoter was conducted, n=4. *, P<0.05 compared to the CTL group. **(f)** A schematic model illustrating potential binding motifs on the ERα promoter, highlighting the Sp1 binding motif (in red) and its mutation (in green). **(g)** Immortalized human HESC cells were infected with either CTL or EZH2 lentivirus (↑EZH2) for 48 hours, followed by transfection with ERα full-length or mutation reporter constructs for a luciferase activity assay, n=5. *P<0.05 vs pERα-2000 group; ¶, P<0.05 vs M-338/Sp1 group. **(h)** Immortalized human HESC cells were infected with either CTL or EZH2 lentivirus (↑EZH2) for 48 hours, followed by ChIP analysis on the ERα promoter, n=4. *P<0.05 vs CTL group. **(i-k)** Immortalized human HESC cells were infected with either CTL or EZH2 knockdown lentivirus (shEZH2) for 48 hours, followed by biological assays. **(i)** mRNA levels were measured by qPCR. **(j)** ChIP analysis for histone 3 methylation on the ERα promoter. **(k)** ChIP analysis of the ERα promoter, n=4. *P<0.05 vs CTL group.

We explored the possible effect of EZH2 on gene expression in immortalized HESC cells. Cells were infected by either control or EZH2-expressing (↑EZH2) lentivirus for 48 hrs, and results showed that EZH2 expression significantly decreased ERα mRNA levels, increased TNFα mRNA, but showed no effect on ERβ mRNA (**Figure 1a**). Similar pattern was observed at the protein level (**Figures 1b, c**; **Supplementary Figure S1a**). To investigate the mechanism of EZH2-mediated ERα suppression, progressive 5’ deletion constructs of the ERα promoter (-2000, -1600, -1200, -800, -600, -400, -300, -200, -100, and 0) were generated and transfected them into HESC cells for luciferase assays. In the presence of EZH2, luciferase activity increased significantly in pERα-300 compared to pERα-2000, further increased in pERα-200, and again in pERα-0, indicating that the EZH2-responsive element lies within -400 to 0 on the ERα promoter (**Figure 1d**). ChIP analysis on this -400 to 0 region revealed that EZH2 expression significantly increased H3K27me3 modification without affecting H3K9me2, H3K9me3, or H3K27me2 (**Figure 1e**). We then analyzed potential binding sites within -400 to 0, mutating each site, which included one GRα and four Sp1 sites (marked in red, **Figure 1f**). Luciferase assays of these mutated constructs indicated that GRα mutation at -159 (M-159/GRα) had no effect, while Sp1 mutations at -338, -303, -227, and -18 increased reporter activity compared to pERα-2000, though less than pERα-0, identifying these Sp1 sites as responsive elements for EZH2-induced ERα suppression. Further ChIP analysis showed that EZH2 expression reduced Sp1 binding but did not affect GRα binding, supporting that EZH2-mediated H3K27me3 modification dissociates Sp1 from the ERα promoter, thus suppressing ERα expression. Finally, we determined the effect of EZH2 knockdown (shEZH2) on ERα expression. shEZH2 treatment increased ERα mRNA levels, decreased TNFα mRNA, and showed no effect on ERβ mRNA compared to CTL (**Figure 1i**). Epigenetic analysis revealed that shEZH2 treatment significantly reduced H3K27me3 modification and increased Sp1 binding at the ERα promoter, without affecting GRα binding (**Figures 1j, k**).

### EZH2 upregulates TNFα expression via decreased ERα and increased ERβ binding on the TNFα promoter

**Figure 2:**
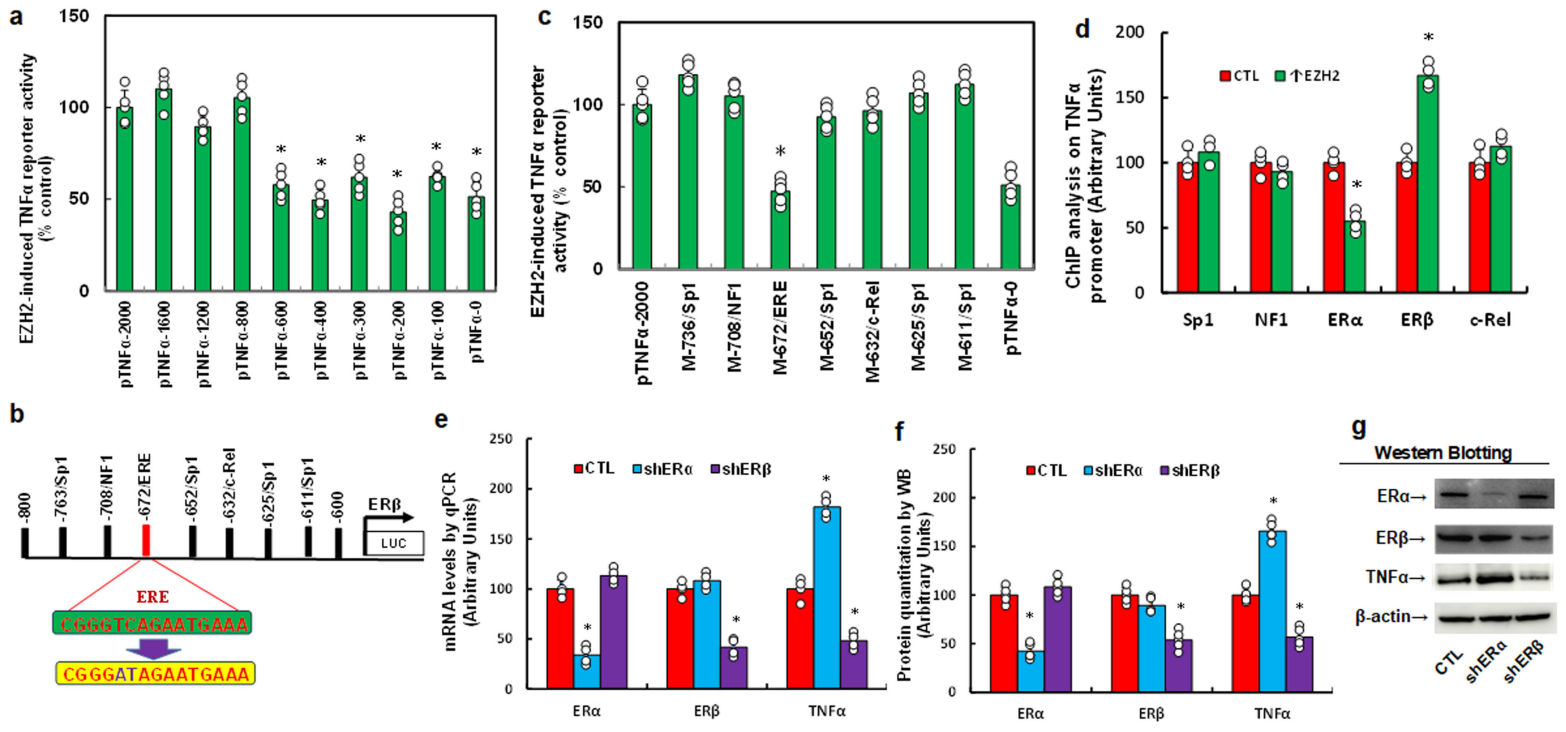
EZH2 enhances TNFα expression by decreasing ERα binding and increasing ERβ binding on the TNFα promoter. **(a-c)** Immortalized human HESC cells were infected with either control (CTL) or EZH2 expression lentivirus (↑EZH2) for 48 hours, followed by transfection with TNFα full-length or deletion reporter constructs for luciferase activity assays, n=5. *P<0.05 vs. pTNFα-2000 group. **(b)** Schematic representation of potential binding motifs on the TNFα promoter, showing the ERE binding motif (in green) and its mutation (in red). **(c)** Immortalized human HESC cells were similarly infected and transfected with TNFα full-length or mutated reporter constructs for luciferase activity assays, n=5. *P<0.05 vs. pTNFα-2000 group. **(d)** Immortalized human HESC cells were infected with either control (CTL) or EZH2 lentivirus (↑EZH2) for 48 hours, followed by ChIP analysis of the TNFα promoter, n=4. *P<0.05 vs. CTL group. **(e-g)** Immortalized human HESC cells were infected with either control (CTL), ERα knockdown (shERα), or ERβ knockdown (shERβ) lentivirus for 48 hours, followed by biological assays. **(e)** mRNA levels measured by qPCR. **(f)** Protein quantification by Western blotting, n=5. **(g)** Representative blots for **(f)**. *P<0.05 vs. CTL group.

To investigate the mechanism behind EZH2-mediated TNFα upregulation in HESC cells, we generated progressive 5’ deletion constructs of the TNFα promoter (-2000, -1600, -1200, -800, -600, -400, -300, -200, -100, and 0) and performed luciferase assays with or without EZH2 overexpression. Results showed a significant decrease in reporter activity with the pTNFα-600 construct compared to pTNFα-2000, with activity remaining constant in subsequent constructs, indicating that the EZH2-responsive element for TNFα regulation lies within -800 to -600 (**Figure 2a**). Within this region, we identified and mutated potential binding sites, including four Sp1 sites, and one site each for NF1, c-Rel, and an estrogen response element (ERE) (marked in red, **Figure 2b**). Luciferase assays revealed that only mutation of the ERE site (M-672/ERE) significantly reduced reporter activity, while other mutations showed no effect, suggesting that EZH2 regulates TNFα via the ERE on its promoter (**Figure 2c**). ChIP analysis further showed that EZH2 expression (↑EZH2) significantly decreased ERα binding and increased ERβ binding to the TNFα promoter (**Figure 2d**). To determine the contributions of ERα and ERβ, we knocked down each receptor individually. ERα knockdown (shERα) significantly increased TNFα mRNA levels, whereas ERβ knockdown (shERβ) significantly decreased TNFα mRNA levels (**Figure 2e**), with protein levels consistent with these mRNA changes (**Figures 2f, g**, **Supplementary Figure S1b**). These results indicate that EZH2 upregulates TNFα by reducing ERα and enhancing ERβ binding on the TNFα promoter. We then explored the role of EZH2 and ERs on TNFα regulation in HEEC cells. EZH2 knockdown (shEZH2) significantly elevated ERα mRNA, reduced TNFα mRNA, but showed no effect on ERβ expression (**Supplementary Figure S2a**). ERα knockdown increased TNFα levels without affecting ERβ, while ERβ knockdown decreased TNFα levels without affecting ERα (**Supplementary Figure S2b**). These results confirm that EZH2 regulates TNFα expression through ERα and ERβ in both HESC and HEEC cells.

### EZH2 inhibitor GSK343 suppresses TNFα expression and increases oxidative stress, mimicking ERβ and EZH2 knockdown effects

**Figure 3:**
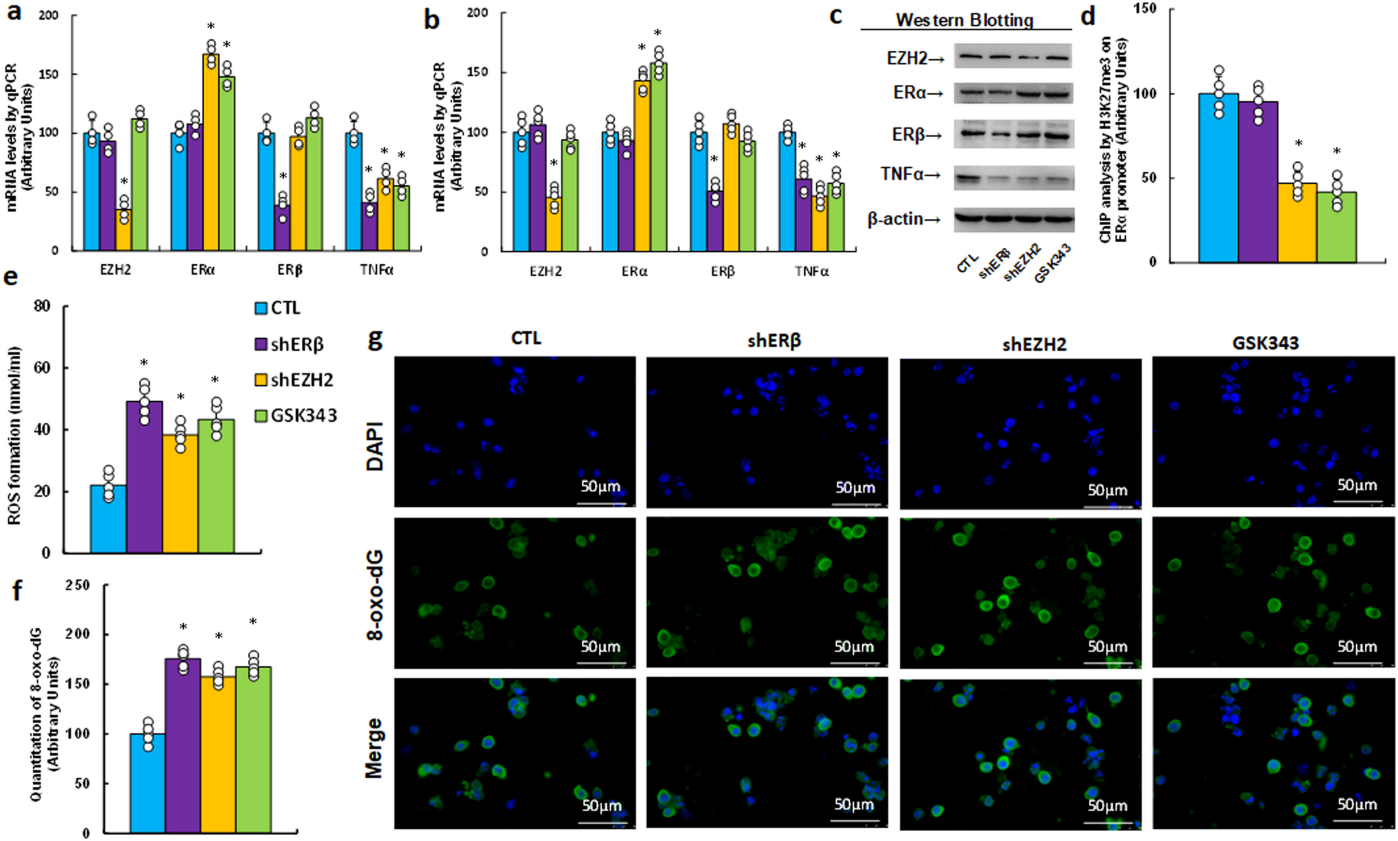
The EZH2 inhibitor GSK343 suppresses TNFα expression and enhances oxidative stress, mimicking the effects of gene knockdown of ERβ and EZH2. HESC cells were treated with control (CTL), knockdown lentivirus for either ERβ (shERβ) or EZH2 (shEZH2), or 5 μM of the EZH2 inhibitor GSK343 for 2 days, after which they were harvested for biological assays. **(a)** mRNA levels were measured by qPCR, n=4. **(b)** Protein quantification was performed using Western blotting, n=5. **(c)** Representative blots for **(b)**. *P<0.05 compared to the CTL group. **(d)** ChIP analysis of H3K27me3 on the ERα promoter, n=5. **(e)** Measurement of reactive oxygen species (ROS) formation. **(f)** Quantification of 8-oxo-dG formation, n=5. **(g)** Representative images for **(f)**. *P<0.05 compared to the CTL treatment.

We explored the impact of the EZH2 inhibitor GSK343 on TNFα expression and redox balance. HESC cells were treated by either control, ERβ knockdown (shERβ), EZH2 knockdown (shEZH2), or GSK343, followed by biological assays. Results showed that shEZH2 treatment increased ERα mRNA, decreased TNFα mRNA, and had no effect on ERβ compared to CTL; shERβ treatment reduced TNFα mRNA without affecting ERα, and GSK343 mirrored the effects of shEZH2 (**Figure 3a**). Protein levels were consistent with mRNA patterns (**Figures 3b, c**, **Supplementary Figure S1c**). Additionally, GSK343 treatment significantly reduced H3K27me3 modification, similar to shEZH2, while shERβ had no effect (**Figure 3d**). In terms of redox balance, GSK343 potentiated ROS production (**Figure 3e**) and 8-oxo-dG formation (**Figures 3f, g**) relative to CTL, mimicking the oxidative stress effects observed with both shERβ and shEZH2 treatments.

### EZH2 inhibitor GSK343 partially reduces pro-inflammatory cytokine release, mimicking ERβ and EZH2 knockdown effects

We assessed the effect of the EZH2 inhibitor GSK343 on pro-inflammatory cytokine release. GSK343 treatment significantly lowered IL1β and TNFα mRNA levels compared with control (CTL) group, reflecting the effects of ERβ (shERβ) and EZH2 (shEZH2) knockdowns (**Supplementary Figure S3a**). Both GSK343 and shEZH2 showed no impact on PGE2 levels, while shERβ significantly reduced PGE2 expression. Additionally, none of the treatments affected IL6 expression. The secretion levels of IL1β (**Supplementary Figure S3b**), IL6 (**Supplementary Figure S3c**), TNFα (**Supplementary Figure S3d**), and PGE2 (**Supplementary Figure S3e**) showed patterns consistent with their mRNA expression, reinforcing the effects observed at the transcript level.

### EZH2 inhibitor GSK343 suppresses cell proliferation, mimicking the effects observed with ERβ and EZH2 gene knockdown

**Figure 4:**
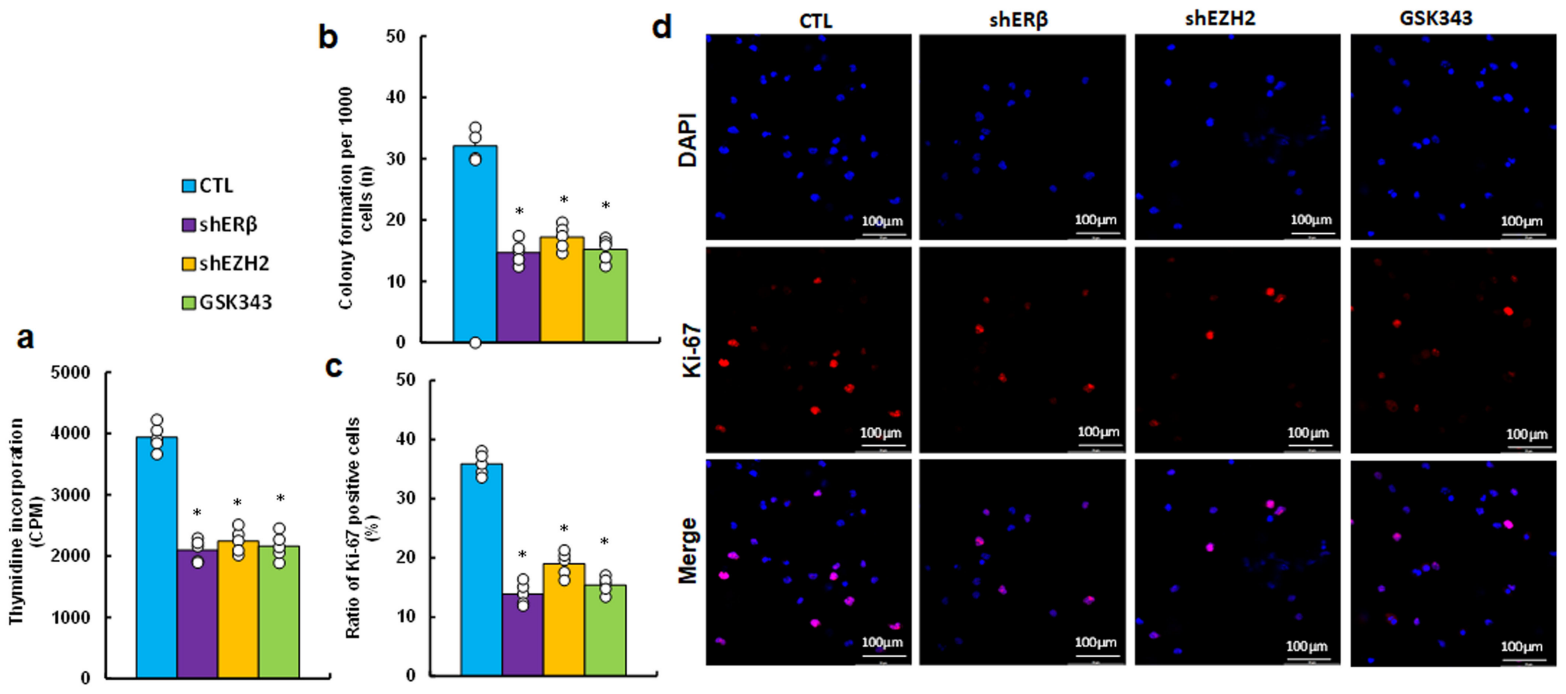
EZH2 inhibitor GSK343 suppresses cell proliferation, similar to the effects observed with ERβ or EZH2 gene knockdown. HESC cells were treated with control (CTL), lentiviral knockdown for ERβ (shERβ) or EZH2 (shEZH2), or 5 μM of EZH2 inhibitor GSK343 for 2 days, followed by biological assays. **(a)** Thymidine incorporation (CPM). **(b)** Colony formation assay. **(c)** Ratio of Ki67-positive cells. **(d)** Representative images for **(c)**. n=5. *P < 0.05 vs. CTL treatment.

We determined the possible effect on cell proliferation and found that EZH2 inhibitor GSK343 significantly suppressed cell proliferation by thymidine incorporation (see **Figure 4a**), colony formation (see **Figure 4b**) and proportion of Ki67-positive cells (see **Figures 4c, d**) compared to CTL group, mimicking the effects observed with gene knockdown of ERβ (shERβ) and EZH2 (shEZH2).

### EZH2 inhibitor GSK343 suppresses inflammation while enhancing oxidative stress, mimicking effects of ERβ and EZH2 knockdown in an endometriosis mouse model

**Figure 5:**
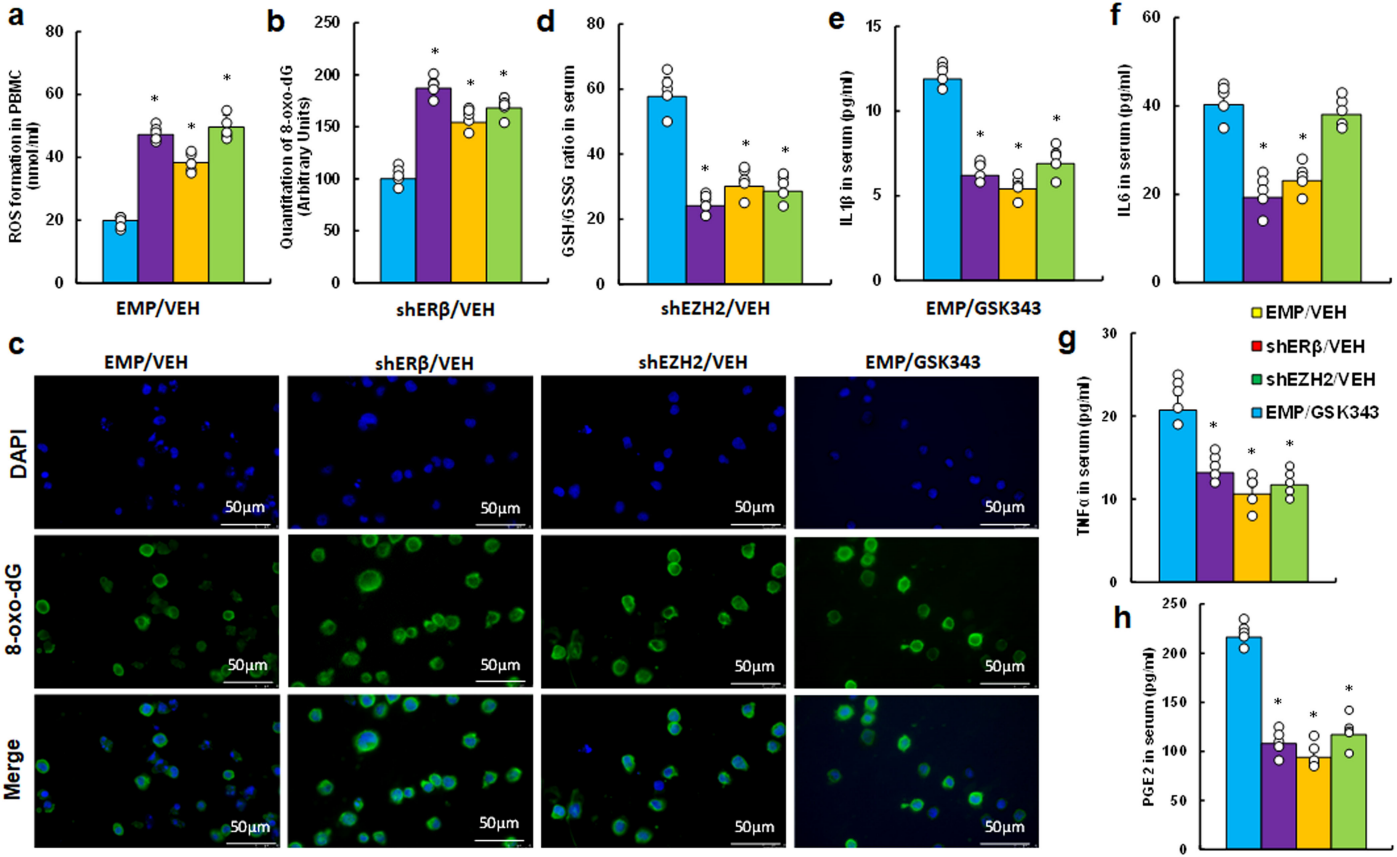
EZH2 inhibitor GSK343 reduces inflammation and increases oxidative stress, paralleling ERβ or EZH2 gene knockdown effects in an endometriosis mouse model. LMP1-treated human HESC or HEEC cells were infected with either empty (EMP) lentivirus or gene knockdown lentivirus for ERβ (shERβ) or EZH2 (shEZH2). A mixture of these treated HESC and HEEC cells was administered intraperitoneally, followed by treatment with vehicle (VEH) or 10 mg/kg GSK343. Serum and PBMCs were isolated for assays. **(a-c)** PBMC analysis: **(a)** ROS formation; **(b)** 8-oxo-dG quantification; **(c)** Representative images for **(b)**. **(d-h)** Serum analysis: **(d)** GSH/GSSG ratio; **(e)** IL1β level; **(f)** IL6 level; **(g)** TNFα level; **(h)** PGE2 level. n=7. *P < 0.05 vs. EMP/VEH group.

We investigated the *in vivo* effects of the EZH2 inhibitor GSK343 on inflammation and redox balance in peripheral blood within an endometriosis mouse model. Our findings indicated that GSK343 treatment (EMP/GSK343) significantly potentiated ROS production (**Figure 5a**) and 8-oxo-dG formation (**Figures 5b, c**) in PBMC cells compared to control (EMP/VEH) group, mirroring the effects seen with ERβ (shERβ) and EZH2 (shEZH2) gene knockdowns. Additionally, serum analysis revealed that GSK343 treatment decreased the proportion of GSH/GSSG relative to EMP/VEH group (**Figure 5d**), consistent with the outcomes of both ERβ and EZH2 knockdown. In addition, We evaluated the secretion of pro-inflammatory cytokines, including IL1β (**Figure 5e**), IL6 (**Figure 5f**), TNFα (**Figure 5g**), and PGE2 (**Figure 5h**). GSK343 significantly reduced serum levels of IL1β, TNFα, and PGE2, aligning with the effects of ERβ (shERβ) and EZH2 (shEZH2) knockdown, though it had minimal impact on IL6 levels. In contrast, gene knockdown of either ERβ or EZH2 significantly lowered IL6 levels compared to the EMP/VEH group.

### EZH2 inhibitor GSK343 enhances oxidative stress and suppresses endometriosis development, mimicking the effects of ERβ or EZH2 gene knockdown in an endometriosis mouse model

**Figure 6:**
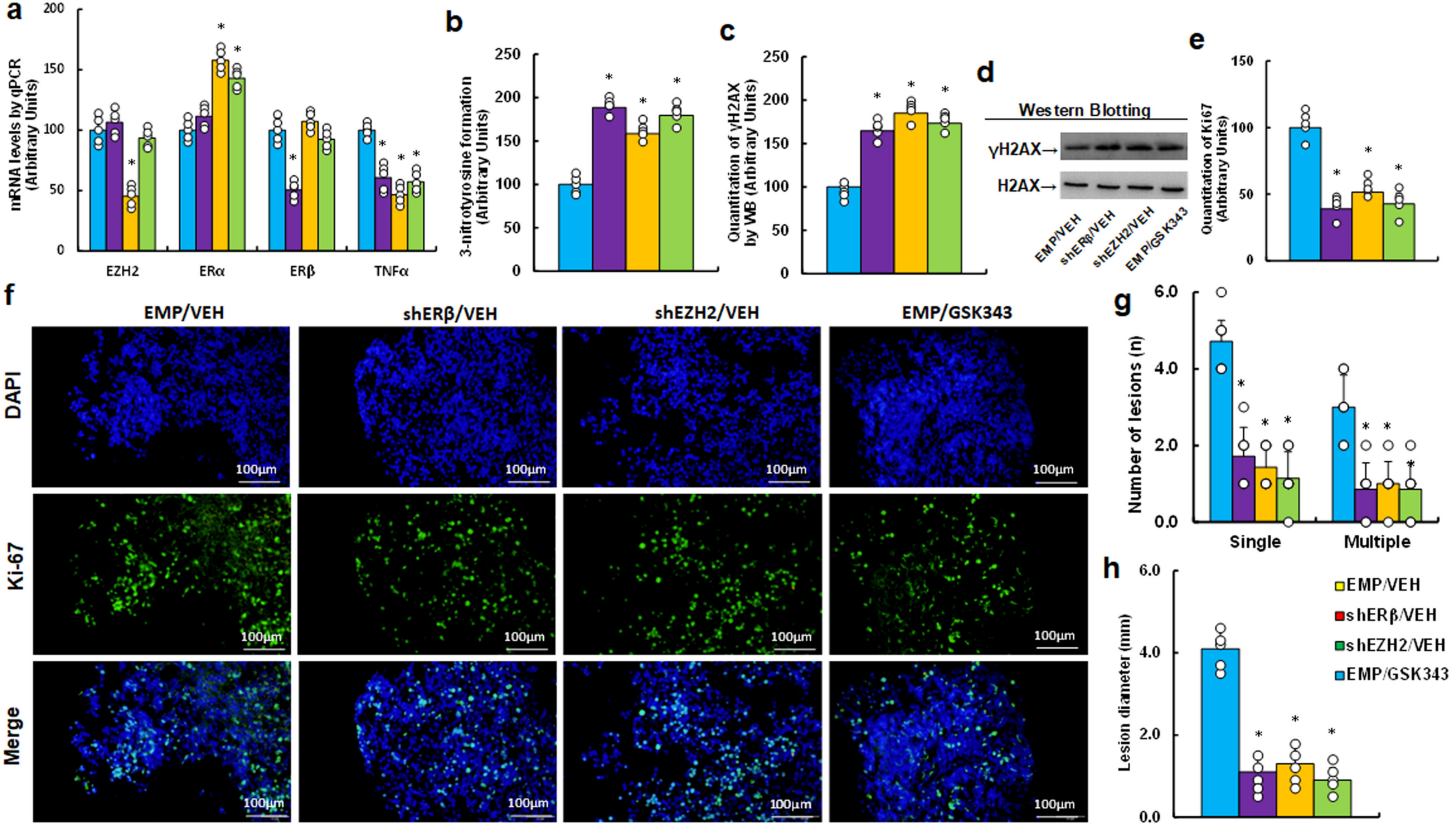
EZH2 inhibitor GSK343 enhances oxidative stress and inhibits endometriosis progression, mimicking ERβ or EZH2 gene knockdown effects in an endometriosis mouse model. LMP1-treated human HESC or HEEC cells were infected with either empty (EMP) lentivirus or gene knockdown lentivirus for ERβ (shERβ) or EZH2 (shEZH2). A mixture of these cells was administered intraperitoneally and treated with vehicle (VEH) or 10 mg/kg GSK343. Endometriosis tissue was then analyzed. **(a)** mRNA levels via qPCR, n=4. **(b)** 3-nitrotyrosine formation. **(c)** Quantification of γH2AX formation. **(d)** Representative western blots for **(c)**. **(e)** Ki67 quantification. **(f)** Representative images for **(e)**. **(g)** Number of lesions, n=7. **(h)** Lesion diameter, n=7. *P < 0.05 vs. EMP/VEH group.

We investigated potential impact of EZH2 inhibitor GSK343 on endometriosis development in a mouse model. Initially, we analyzed mRNA expression in endometriosis lesions and found that shEZH2 treatment (EMP/GSK343) increased ERα mRNA while decreasing TNFα mRNA, with no effect on ERβ compared to EMP/VEH group. In contrast, shERβ treatment reduced TNFα mRNA without affecting ERα levels, and GSK343 mimicked the effects of shEZH2 (see **Figure 6a**). Next, we examined the effects on redox balance and found that GSK343 significantly enhanced 3-nitrotyrosine generation (see **Figure 6b**) and γH2AX generation (see **Figures 6c, d**; **Supplementary Figure S1d**) compared to EMP/VEH group. Additionally, Ki67 immunohistochemistry (IHC) staining revealed that GSK343 significantly reduced Ki67 expression compared to EMP/VEH group (see **Figures 6e, f**). Finally, we assessed lesion sizes and found that GSK343 significantly decreased both lesion numbers (single and multiple nodules) (see **Figure 6g**) and lesion size (see **Figure 6h**) compared to EMP/VEH group. All these effects were similarly observed with gene knockdown of either ERβ (shERβ) or EZH2 (shEZH2).

### Altered gene expression of EZH2, estrogen receptors, TNFα, and oxidative stress in human endometriosis subjects

**Figure 7:**
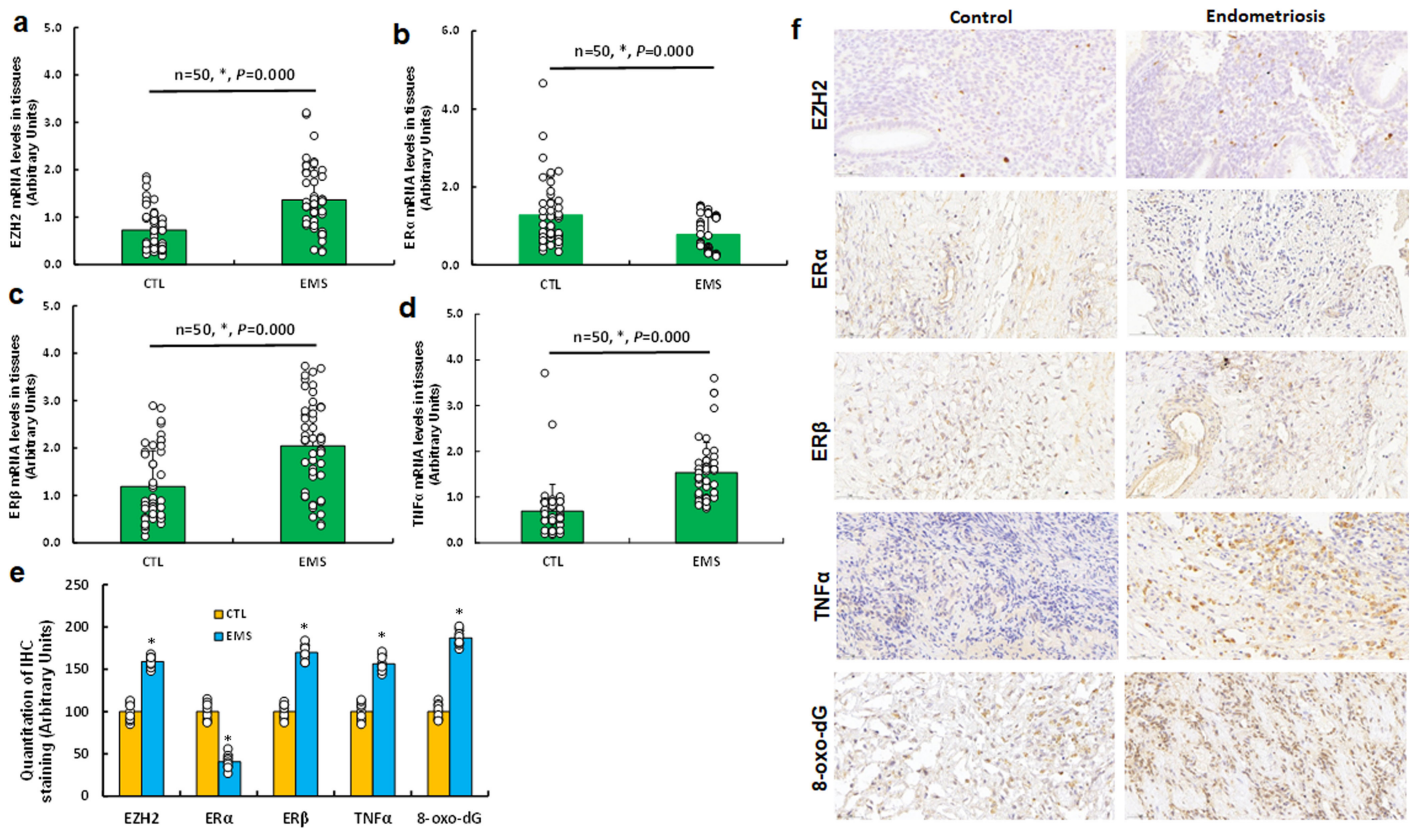
Differential gene expression of EZH2, estrogen receptors, TNFα, and oxidative stress markers in human endometriosis subjects. Tissue samples were collected from 50 control (CTL) or endometriosis (EMS) participants for biological assays. **(a-d)** qPCR mRNA analysis of EZH2 **(a)**, ERα **(b)**, ERβ **(c)**, and TNFα **(d)**, n=50. **(e)** Quantification of IHC staining, n=7. **(f)** Representative images for **(e)**. *P < 0.05 vs. CTL group.

We analyzed 50 cases of human subjects, categorized as either control (CTL) or endometriosis (EMS), following surgical intervention. We assessed the mRNA levels of EZH2 (see **Figure 7a**), ERα (see **Figure 7b**), ERβ (see **Figure 7c**), and TNFα (see **Figure 7d**) in endometriosis lesions. Our findings indicated that EMS samples exhibited increased mRNA levels of EZH2, ERβ, and TNFα, while ERα levels were significantly decreased compared to CTL group. Subsequently, we determined protein levels through immunohistochemistry (IHC) staining, which revealed a similar expression pattern to that of mRNA level. Notably, the EMS group displayed significantly higher levels of 8-oxo-dG staining compared to CTL group (see **Figures 7e, f**).

### Schematic model of EZH2-induced endometriosis development via estrogen receptor and TNFα expression

**Figure 8:**
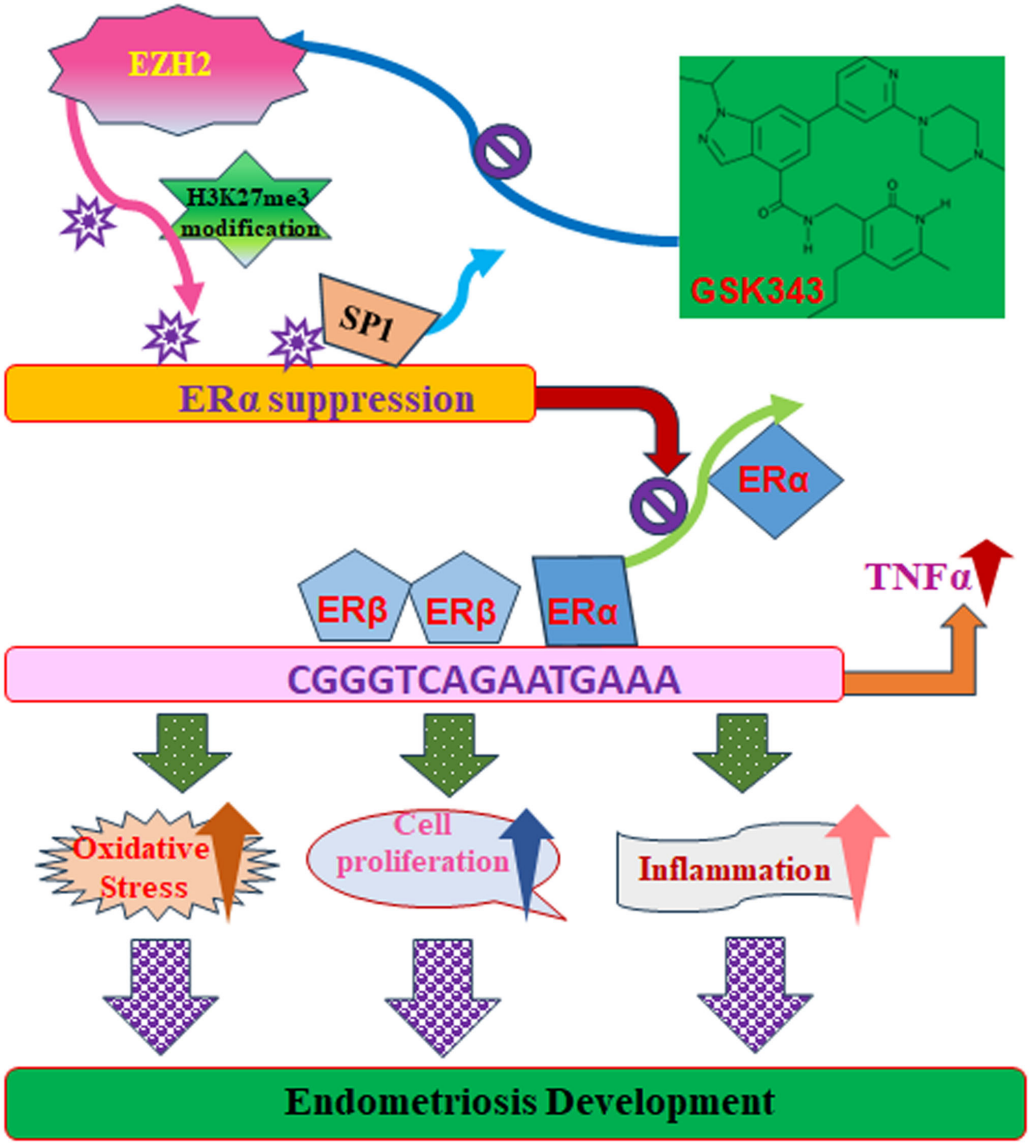
Proposed model for EZH2-mediated endometriosis development through modulation of estrogen receptor and TNFα expression. ER, estrogen receptor; EZH2, enhancer of zeste homolog 2; H3K27me3, histone H3 lysine 27 trimethylation; Sp1, specificity protein 1; TNFα, tumor necrosis factor α.

We developed a schematic model for EZH2-mediated endometriosis progression. Increased EZH2 expression leads to elevated H3K27me3 modifications, disrupting Sp1 binding at the ERα promoter and subsequently downregulating ERα expression. Reduced ERα binding to the TNFα promoter enhances ERβ binding at this site, as both estrogen receptors compete for the same estrogen-responsive element on the TNFα promoter. This shift triggers TNFα upregulation, leading to oxidative stress, cell proliferation, inflammation, and ultimately promoting endometriosis progression. Conversely, the EZH2 inhibitor GSK343 reduces EZH2-driven H3K27me3 modifications, thereby suppressing TNFα levels and impeding endometriosis development (see **Figure 8**).

## Discussion

In this research, we found that EZH2 suppresses ERα expression via H3K27me3 modification and subsequent dissociation of Sp1 from the ERα promoter. Additionally, EZH2 indirectly upregulates TNFα expression by decreasing ERα binding and increasing ERβ binding to the TNFα promoter in *in vitro* cell experiments. The EZH2 inhibitor GSK343 was shown to suppress TNFα expression and endometriosis development in a mouse model. Furthermore, our study of human subjects revealed increased expression of EZH2, ERβ, and TNFα, alongside decreased ERα levels in EMS lesions compared to control group.

### EZH2 and estrogen receptors

Our findings indicate that EZH2 enhances H3K27me3 modification on the ERα promoter, resulting in the suppression of ERα expression while having no significant effect on ERβ expression in the *in vitro* experiments (10). Moreover, ERα knockdown did not significantly alter ERβ levels. In the context of endometriosis, ERβ expression is frequently upregulated, whereas ERα levels are reduced compared to normal endometrial tissue. This altered ratio is a hallmark of the disease and contributes to the proliferative and inflammatory responses observed in endometriotic lesions. While EZH2 typically represses gene expression, some studies suggest that in endometriosis, the balance of ER expression (favoring ERβ over ERα) may be influenced by epigenetic regulators like EZH2 (32, 33). Notably, our human study revealed that EMS lesions exhibited increased EZH2 expression alongside suppressed ERα and upregulated ERβ expression compared to the control group. This suggests an EZH2-independent mechanism for ERβ upregulation in EMS development, potentially involving epigenetic changes (34, 35), the IL1β inflammatory signaling pathway (36), SIRT1 (37), and viral predisposition (21).

### EZH2 and inflammation

Our *in vitro* experiments uncovered a novel mechanism by which EZH2 mediates TNFα upregulation through decreased ERα binding and increased ERβ binding to the TNFα promoter, as both estrogen receptors compete for binding to the estrogen response element. Estrogen receptors are known to regulate several key inflammation-related genes, including IL6, IL1β, and TNFα (38, 39). Our results indicate that EZH2-mediated H3K27me3 modification suppresses ERα expression while leaving ERβ expression unaffected, ultimately leading to TNFα upregulation. Interestingly, knockdown of EZH2 (shEZH2) was found to suppress TNFα and IL1β, with no effect on IL6 or PGE2. This suggests that estrogen receptors may regulate inflammation through direct effects on ER expression or via other indirect non-genomic signaling pathways (40, 41).

### EZH2 and endometriosis development

EZH2 has the capacity to suppress multiple genes through H3K27me3 modification, and its expression is reported to be upregulated in endometriotic lesions (10, 11). Our results show that EZH2 suppresses ERα expression while upregulating TNFα expression, thereby enhancing inflammation and cell proliferation in *in vitro* experiments. The EZH2 inhibitor GSK343 (10, 11) effectively suppressed EMS development in a mouse model, mimicking the effects of gene knockdown of ERβ and EZH2. Additionally, our human study demonstrated increased EZH2 expression in endometriotic lesions, accompanied by upregulation of ERβ and TNFα, as well as suppression of ERα, indicating that EZH2 plays a significant role in endometriosis development.

### Effect of GSK343 on endometriosis development

We conducted a preliminary dose-response study using GSK343 at 5, 10, 15, and 20 mg/kg. The 5 mg/kg dose showed minimal efficacy, while 10 mg/kg produced a clear therapeutic effect without observable toxicity. Higher doses (15 and 20 mg/kg) resulted in increased toxicity, particularly at 20 mg/kg. Based on these findings, we selected 10 mg/kg as the optimal dose for balancing efficacy and safety in our mouse model.

Our data indicate that GSK343 increases oxidative stress markers (e.g., ROS, 8-oxo-dG) while reducing inflammatory cytokines. Oxidative stress plays a dose-dependent role in endometriosis: moderate ROS promotes cell survival and lesion progression, whereas excessive ROS- as induced by GSK343- can overwhelm antioxidant defenses, leading to DNA damage and cell death (42, 43). This dual effect may explain GSK343’s inhibitory impact on lesion growth despite a reduction in inflammatory signals. Thus, GSK343 appears to exert both pro-oxidative and anti-inflammatory actions that contribute to the disruption of EMS pathology. Additionally, our results show that GSK343 partially reverses EZH2-mediated H3K27me3 modifications, although these marks remain elevated compared to controls for at least two days post-treatment. This persistence indicates that while GSK343 can modulate the epigenetic landscape, some changes may be sustained over time (44), which is a critical consideration when developing therapeutic strategies for chronic conditions like endometriosis.

## Conclusions

In summary, EZH2-mediated H3K27me3 modification suppresses ERα expression while enhancing TNFα expression. The EZH2 inhibitor GSK343 effectively suppresses inflammation and EMS development in a mouse model, mimicking the effects of gene knockdown of ERβ and EZH2. Increased EZH2 expression, alongside upregulated TNFα and suppressed ERα in human endometriotic lesions, further supports the critical role of EZH2 in EMS development. Targeting EZH2 may offer potential therapeutic strategies for managing inflammation and associated symptoms in endometriosis.

## Data Availability

The original contributions presented in the study are included in the article/**Supplementary Material**. Further inquiries can be directed to the corresponding authors.
