# Supplementary material for: EZH2 promotes endometriosis progression through estrogen receptor and TNFα expression
Source: Front Endocrinol (Lausanne). 2025 Jun 24;16:1574938. doi: 10.3389/fendo.2025.1574938 (PMC12234293; doi:10.3389/fendo.2025.1574938)
Supplement: Supplementary file 1 [file Table1.docx]

**EZH2 Promotes Endometriosis Progression through Estrogen Receptor and TNFα Expression**

Xiaohan Liu^1,^*, Liqin Cheng^1,^*, Liuxuan Huang^1^, Mingyue Li^1^, Qingjun Shen^1^, Donghan Li^1^,

Kailing Dai^1^, Yanxia Fu^1^, Min Li^2^, Paul Yao^1,#^, Liqin Zeng^1,#^

**Supplementary Materials**

**Data S1: Materials and Methods**

**Reagents and Materials**: The human endometrial epithelial cells (HEEC, #PRI-H-00048) and human endometrial stromal cells (HESC, #PRI-H-00098) were purchased from ZQXZ Bio (Shanghai, China), and cultured in completed medium of PCM-H-068 HEEC Medium Kit and PCM-H-120 HESC Medium Kit, respectively (from ZQXZ Bio), and they were confirmed by positive immunostaining of CK19 and Vimentin, respectively, and passage 3 was used in this study. In some experiments, both HEEC and HESC cells were conditionally immortalized using a hTERT lentivirus vector with an extended life span to achieve higher transfection efficiency and experimental stability (1). All cells were maintained in a humidified incubator with 5% CO_2_ at 37°C. 24 h before treatments, the medium was changed by phenol red-free DMEM supplemented with charcoal-stripped FBS. 17β-estradiol (E2, #E2758) and EZH2 inhibitor GSK343 (#SML0766, dissolved in 0.1% DMSO) were purchased from Sigma, China (2). The EBV LMP1 adenovirus for LMP1 transient infection and related empty control adenovirus were prepared previously in our lab (3).

Antibodies for β-actin (sc-47778), ERα (sc-8005), ERβ (sc-137381), c-Rel (sc-6955), Ki-67 (sc-101861), NF1 (sc-74444), SOD2 (sc-30080), Sp1 (sc-17824) and TNFα (sc-52746) were obtained from Santa Cruz Biotechnology. Antibodies for EZH2 (ab186006), H3K9me2 (ab1220), H3K9me3 (ab8898), H3K27me2 (ab24684), H3K27me3 (ab6002), H2AX (ab20669), γH2AX (ab2893) were obtained from Abcam. The antibody for 8-oxo-dG (4354-MC-050) was obtained from Novus Biologicals. Nuclear extracts were prepared using the NE-PER Nuclear and Cytoplasmic Extraction Reagents Kit (Pierce Biotechnology). Protein concentration was measured using the Coomassie Protein Assay Kit (Pierce Biotechnology) per manufacturers’ instructions.

**Construction of reporter plasmids**. In order to construct human ERα**/**TNFα reporter plasmids, the promoter (2kb upstream of the transcription start site plus first exon) was amplified from Ensembl ID: ESR1-201 ENST00000206249.8 (for ERα) and TNF-201 ENST00000376122.3 (for TNFα) by PCR from human genomic DNA and subcloned into the pGL3-basic vector (#E1751, Promega) using underlined restriction sites with the following primers: ERα forward: 5’-gcgc-ggtacc- cac aca ctc tct ctg cct agt -3’ (Kpn1) and ERα reverse: 5’- gtac- ctcgag- ctg tag aat gcc ggc ggg ccg -3’ (Xho1); TNFα forward: 5’-gcgc-ggtacc-gca ctc gat gta cca cgg ggc -3’ (Kpn1) and TNFα reverse: 5’- gtac- ctcgag- ctc ttc cct ctg ggg gcc gat -3’ (Xho1). To map promoter activity, the related deletion promoter constructs were generated by PCR methods and subcloned into the pGL3-basic vector.

**Preparation of human EZH2 expression lentivirus***.* The cDNA for human EZH2 (obtained from Open Biosystems) was subcloned into the pLVX-Puro vector (from Clontech) with the restriction sites of Xho1 and BamH1 using the following primers: EZH2 forward primer: 5’- gtac - ctcgag (Xho1) - atg ggc cag act ggg aag aaa -3’ and EZH2 reverse primer: 5’- gtac - ggatcc (BamH1) - tca agg gat ttc cat ttc tct -3’. The lentivirus for either EZH2 or empty control (CTL) was expressed through Lenti-X™ Lentiviral Expression Systems (from Clontech) per manufacturers’ instructions.

**Preparation of shEZH2 knockdown lentivirus**. The shRNA lentivirus plasmid for human EZH2 (sc-35312-SH) or non-target control (sc-108060) was purchased from Santa Cruz Biotechnology. The related lentivirus for shEZH2 or empty control (CTL) was expressed through Lenti-X™ Lentiviral Expression Systems (from Clontech) per manufacturers’ instructions. The purified and condensed lentivirus was used for *in vitro* gene knockdown, and knockdown efficiency was confirmed by mRNA reduction of more than 65% compared to the control group using real time PCR (see Table S1).

**RT reaction and real-time quantitative PCR.** Total RNA from treated cells was extracted using the RNeasy Micro Kit (Qiagen), and the RNA was reverse transcribed using an Omniscript RT kit (Qiagen). All the primers were designed using Primer 3 Plus software with the Tm at 60°C, primer size of 21 bp, and product length in the range of 140-160 bp (see Table S1). The primers were validated with an amplification efficiency in the range of 1.9-2.1 and the amplified products were confirmed with agarose gel. Real-time quantitative PCR was run on iCycler iQ (Bio-Rad) using the Quantitect SYBR green PCR kit (Qiagen). The PCR was performed by denaturing at 95°C for 8 min followed by 45 cycles of denaturation at 95°C, annealing at 60°C, and extension at 72°C for 10 s, respectively. 1 µl of each cDNA was used to measure target genes. β-actin was used as the housekeeping gene for transcript normalization, and the mean values were used to calculate relative transcript levels with the ^ΔΔ^CT method per instructions from Qiagen. In brief, the amplified transcripts were quantified by the comparative threshold cycle method using β-actin as a normalizer. Fold changes in gene mRNA expression were calculated as 2^−ΔΔCT^ with CT = threshold cycle, ΔCT=CT (target gene)-CT(β-actin), and the ΔΔCT =ΔCT (experimental)-ΔCT (reference) (4, 5).

**Luciferase reporter assay.** Cells were seeded in a 6-well plate and cultured in complete medium until 80% confluent. Cells were then co-transfected by 3 µg of related reporter plasmid as well as 0.2 µg of pRL-CMV-Luc *Renilla* plasmid (from Promega) for internal transfection efficiency control. 24 hours after transfection, cells were further treated as indicated and then harvested. Luciferase activity was determined using Dual-Luciferase^TM^ Assay System (Promega) as per manufacturers’ instructions. The transfection efficiencies were calculated accordingly using the *Renilla* plasmid and the reporter activities were normalized and calculated.

**Chromatin immunoprecipitation (ChIP).** Cells were washed and crosslinked using 1% formaldehyde for 20 min and terminated by 0.1M glycine. Cell lysates were sonicated and centrifuged. 500 µg of protein were pre-cleared by BSA/salmon sperm DNA with preimmune IgG and a slurry of Protein A Agarose beads. Immunoprecipitations were performed with the indicated antibodies, BSA/salmon sperm DNA and a 50% slurry of Protein A agarose beads. Input and immunoprecipitates were washed and eluted before then being incubated with 0.2 mg/ml Proteinase K for 2 h at 42˚C, followed by 6 h at 65˚C to reverse the formaldehyde crosslinking. DNA fragments were recovered through phenol/chloroform extraction and ethanol precipitation. A ~150 bp fragment on the related promoter was amplified by real-time PCR (qPCR) using the primers provided in Table S1 (4, 5).

**Western blotting.** Cells were lysed in an ice-cold lysis buffer (0.137M NaCl, 2mM EDTA, 10% glycerol, 1% NP-40, 20mM Tris base, pH 8.0) with protease inhibitor cocktail (Sigma). The proteins were separated in 10% SDS-PAGE and transferred to the PVDF membrane, which was then blotted using primary antibodies (1:1000) and then simultaneously incubated with the differentially labeled species-specific secondary antibodies, anti-RABBIT IRDye™ 800CW (green) and anti-MOUSE (or goat) ALEXA680 (red). Membranes were scanned and quantitated using the ODYSSEY Infrared Imaging System (LI-COR, NE) (6).

**Immunostaining**. The treated cells were transferred to cover slips coated with 0.1% gelatin, fixed by 3.7% formaldehyde at 37ºC for 15 min, permeabilized by 1% BSA+0.2% Triton X-100 in PBS for 1 hour, then blotted with 40 μg/ml (dilute 1:50) of either Ki-67 or 8-oxo-dG monoclonal antibodies for 2 hours. Cells were then washed three times and the FITC/Texas Red labeled anti-mouse/rabbit secondary antibody (1:100) was added for blotting for another 1 hour. After thorough washing, the slides were visualized and photographed; cell nuclei were stained with 4’,6-diamidino-2-phenylindole dihydrochloride (DAPI, #D9542, from Sigma) and the staining was quantitated by Image J.

**Measurement of oxidative stress.** Treated cells were seeded in a 24-well plate and incubated with 10 μM CM-H2DCFDA (Invitrogen) for 45 min at 37°C; intracellular formation of reactive oxygen species (ROS) was then measured at excitation/emission wavelengths of 485/530nm using a FLx800 microplate fluorescence reader (Bio-Tek), and the data was normalized as arbitrary units (5, 7). The GSH/GSSG ratio was measured using the GSH/GSSG-Glo™ Assay Kit (#V6611, obtained from Promega) per manufacturers’ instructions. 3-nitrotyrosine (3-NT) was measured using a 3-Nitrotyrosine ELISA Kit (#ab116691 from Abcam) according to manufacturers’ instructions. The formation of γH2AX was measured from nuclear extracts by western blotting using H2AX as the input control (5).

**Analysis of cytokines**. Human cytokines, including IL1β, IL6, and TNF-α from in vitro cell culture supernatant, were measured using Human IL-1 beta/IL-1F2 Quantikine ELISA Kit (#DLB50), Human IL-6 Quantikine ELISA Kit (#D6050) and Human TNF-alpha Quantikine ELISA Kit (#DTA00D), respectively; Mouse cytokine secretion, including IL1β, IL6 and TNFα, were measured by Mouse IL-1β/IL-1F2 Quantikine ELISA Kit (#MLB00C), Mouse IL-6 Quantikine ELISA Kit (#M6000B) and Mouse TNF-alpha Quantikine ELISA Kit (#MTA00B), respectively; and the PGE2 was measured by [Prostaglandin E2 Parameter Assay Kit](https://www.rndsystems.com/products/prostaglandin-e2-parameter-assay-kit_kge004b) (#[KGE004B](https://www.rndsystems.com/products/prostaglandin-e2-parameter-assay-kit_kge004b)) according to manufacturers’ instructions from R&D Systems (8).

**DNA synthesis by [^3^H]-thymidine incorporation**. Cell proliferation was evaluated as the rate of DNA synthesis by [^3^H]-methylthymidine incorporation (9). Cells were pooled in 24-well plates until they reached 80% confluence. After treatment, cells were incubated with serum-free media containing ^3^H-methylthymidine (0.5 µCi/well) for 2 hours and then washed twice with PBS. Cellular DNA was precipitated using 10% trichloroacetic acid and solubilized with 0.4M NaOH (0.5 ml/well). Incorporation of ^3^H-methylthymidine into the DNA was measured in a scintillation counter and was determined as counts per minute (CPM) (5).

**Colony formation in soft agar**. This assay is a method for evaluating the ability of individual cell lines to grow in an anchorage-independent manner. Cells were resuspended in DMEM containing 5% FBS with 0.3% agarose and layered on top of 0.5% agarose in DMEM on 60mm plates. 1000 cells were seeded in 60mm soft agar dishes for 30 days. The dishes were examined twice per week, and colonies that grew beyond 50mm in diameter were scored as positive. Each experiment was done in quadruplicate (5).

**In vivo mouse experiments**. The NOD scid gamma (NSG) mouse was purchased from Jackson lab. All procedures involving mice were conducted in accordance with NIH regulations concerning the use and care of experimental animals and were approved by the Institutional Animal Care and Use Committee of Sun Yat-Sen University Affiliated No.8 Hospital. All the female mice were housed 4 or 5 per cage on a 12:12-h light-dark cycle and were given commercial rodent chow and water ad libitum on arrival. All animal handling was carried out under laminar-flow hoods, and all the invasive procedures were performed with the animals under isoflurane inhalation anesthesia or a cocktail of ketamine (100mg/kg) and xylazine (10mg/kg) intraperitoneally. At 4 weeks of age, the female mice received bilateral ovariectomy (OVX) surgery. After one week of surgery, all the NSG mice were implanted subcutaneously with sterile 60-day release pellets containing 0.72mg of 17β-estradiol (E2, Innovative Research of America, Shanghai China) via a ~3-mm incision on the dorsal aspect of the neck. After 2 days of E2 pellets administration, the mixed human endometrial cells were transplanted as described below:

Cell transplantation and treatment. Both HESC and HEEC cells were infected by LMP1 adenovirus for 48 hours, then the adenovirus was removed and cultured continuously to pick up the single colony until passage 6 (3), then the cells were infected by either empty control (EMP), shEZH2 or shERβ lentivirus for later transplantation. On the day of transplantation, the above treated cells were trypsinized and 2×10^6^ of cells for both HESC and HEEC were mixed, pelleted and washed, then resuspended and mixed with Matrigel (BD Biosciences, Beijing China) at a 1:1 ratio in a final volume of 150µl, and maintained on ice until transplantation. The suspension mixtures were loaded in 1ml insulin syringe fitted with 20-gauge needle, and administered intraperitoneally on the midventral line just caudal to the umbilicus without any damage to the peritoneal layer or organs. Immediately after transplantation, the mice were kept to sternal posture to facilitate attachment of cells with peritoneum (10). After 24 hours of cell transplantation, the mice were randomly divided into below 4 groups. Group 1: mice received empty lentivirus treated cells (EMP) and vehicle control injection (EMP/VEH); Group 2: mice received shERβ treated cells and vehicle control injection (shERβ/VEH); Group 3: mice received shEZH2 treated cells and vehicle control injection (shEZH2/VEH); Group 4: mice received empty lentivirus treated cells (EMP) and 10mg/kg body weight of GSK343 that was given as gavage every 2 days for 4 weeks (EMP/GSK343).

Assessment and characterization of endometriosis lesions. After 4 weeks of cell transplantation, the recipient NSG mice were euthanized by CO2 asphyxiation, the whole blood was collected by heart puncture, then the serum and peripheral blood mononuclear cells (PBMC) were isolated. The abdominal cavity was opened and the presence of endometriosis lesions was examined by gross visual examination. The number of endometriosis lesions was counted using a dissection microscope, and the size of each lesion was measured with a caliper. The lesions were categorized into single and multiple, where single lesions consisted of 1 visible nodule, and multiple lesions consisted of 2 or more visible nodules (10, 11). The PBMC was used to determine redox balance, and the serum was used for measurement of GSH/GSSG ratio and the secretion of proinflammatory cytokines. Part of the lesions were used for gene expression through real time PCR for mRNA and Western Blotting for protein levels (12). Immediately, the peritoneum was fixed in 4% paraformaldehyde, and the entire peritoneum was sectioned for immunohistochemistry staining (10).

**Isolation of mouse PBMC cells**. Heparinized peripheral blood was collected from mouse subjects by heart puncture and diluted 1:3 with Hank's balanced salts solution without Ca^2+^/Mg^2+^ (HBSS solution). The diluted blood was layered onto 10ml of Ficoll-Paque in 15ml sterile centrifuge tubes followed by centrifugation at 300×g at 20ºC for 40min. The PBMC layers were then harvested and washed by HBSS solution. The pellets were resuspended with lysing buffer containing 150 mM NH4Cl, 1.0 mM KHCO3, and 0.1 mM Na2EDTA, pH 7.4 and incubated for 5 min at room temperature to remove contaminated red cells. The cell suspensions were then centrifuged and washed with HBSS solution before the cell pellet was resuspended for further biological assay.

**Immunohistochemistry (IHC).** The endometriotic lesions were cut into 10µm sections under Cryostat Microtome, and fixed in 2% paraformaldehyde for 15 min at room temperature and then fixed in methanol for 10 min at 4°C before being permeabilized by 1% BSA+0.2% Triton X-100 in PBS, and then blotted by 40μg/ml of antibodies for ERα, ERβ, EZH2, Ki67, TNFα or 8-oxo-dG for 2 hours. After another 1 hour of blotting by FITC labeled anti-mouse/rabbit secondary antibodies (for animal tissues), sections were mounted under coverslips using fluorescent mounting media with 46-diamidino-2-phenylindole (DAPI). On the other hand, the slides were blotted by HRP-labeled anti-mouse/rabbit antibodies, and stained by diaminobenzidine (DAB) (for human tissues). The slides were visualized and photographed, and the protein expression (60 cells in each group) were quantitated by Image J. software (11, 13).

**Human subjects study**. The human study was reviewed and approved by the Institutional Ethical Committee from The Eighth Affiliated Hospital of Sun Yat-Sen University. Written consent was obtained from each participant for laparoscopy or laparotomy with benign indications including uterine bleeding, pelvic pain, elective contraception, infertility, or endometriosis. Inclusion criteria included women who were aged 18-49 years, had regular menstrual cycles, and used no hormonal therapy for at least 3 months prior to surgery. Exclusion criteria included post-menopausal status, previous hormone use within 3 months of surgery, hyperplasia, polyps, malignancy, autoimmune disease, cardiovascular disease, or use of anti-inflammatory medications. 50 subjects were included in each group: endometriosis patients and controls with no evidence of endometriosis or endometrial pathology. Endometrial biopsies in the secretory phase were obtained from women with moderate-to-severe disease (American Society for Reproductive Medicine stages III and IV). The endometriotic group was surgically diagnosed and histologically verified and the control group patients were visually verified to be free of endometriosis during the surgery. Controls underwent surgery for benign gynecologic disease and had no evidence of endometriosis. The most common indications for surgery in the controls were elective tubal ligation, infertility, uterine bleeding or ovarian cysts. The phase of the menstrual cycle was determined based on the subjects’ menstrual history and last menstrual period (14). The tissues were collected for the analysis of mRNA levels by qPCR or immunohistochemistry for staining of ERα, ERβ, EZH2, TNFα and 8-oxo-dG. The participants’ clinical data and demographic characters are shown in Table 1.

**Statistical analysis.** The data was given as mean ± SD; all of the experiments were performed at least in quadruplicate unless otherwise indicated. The data was analyzed as normal distribution using Shapiro-Wilk test to evaluate the normality of the data (15). Comparisons were made on each variable using χ^2^ tests, the unpaired Student’s t-tests or one-way analysis of variance (ANOVA) followed by the Tukey−Kramer test to determine statistical significance of different groups using SPSS 22 software, and a *P* value < 0.05 was considered significant (5).

REFERENCES

1. Kong D, Zhan Y, Liu Z, Ding T, Li M, Yu H, et al. SIRT1-mediated ERβ suppression in the endothelium contributes to vascular aging. *Aging Cell.* 2016;15(6):1092-102.

2. Del Moral-Morales A, Gonzalez-Orozco JC, Hernandez-Vega AM, Hernandez-Ortega K, Pena-Gutierrez KM, and Camacho-Arroyo I. EZH2 Mediates Proliferation, Migration, and Invasion Promoted by Estradiol in Human Glioblastoma Cells. *Front Endocrinol (Lausanne).* 2022;13:703733.

3. Wang J, Liang Y, Liang X, Peng H, Wang Y, Xu M, et al. Evodiamine suppresses endometriosis development induced by early EBV exposure through inhibition of ERbeta. *Front Pharmacol.* 2024;15:1426660.

4. Zou Y, Lu Q, Zheng D, Chu Z, Liu Z, Chen H, et al. Prenatal levonorgestrel exposure induces autism-like behavior in offspring through ERbeta suppression in the amygdala. *Mol Autism.* 2017;8:46.

5. Zhang H, Li L, Li M, Huang X, Xie W, Xiang W, et al. Combination of betulinic acid and chidamide inhibits acute myeloid leukemia by suppression of the HIF1alpha pathway and generation of reactive oxygen species. *Oncotarget.* 2017;8(55):94743-58.

6. Ceradini DJ, Yao D, Grogan RH, Callaghan MJ, Edelstein D, Brownlee M, et al. Decreasing intracellular superoxide corrects defective ischemia-induced new vessel formation in diabetic mice. *J Biol Chem.* 2008;283(16):10930-8.

7. Yao D, Shi W, Gou Y, Zhou X, Yee Aw T, Zhou Y, et al. Fatty acid-mediated intracellular iron translocation: a synergistic mechanism of oxidative injury. *Free Radic Biol Med.* 2005;39(10):1385-98.

8. Kobayashi EH, Suzuki T, Funayama R, Nagashima T, Hayashi M, Sekine H, et al. Nrf2 suppresses macrophage inflammatory response by blocking proinflammatory cytokine transcription. *Nat Commun.* 2016;7:11624.

9. Somasundaram K, and El-Deiry WS. Inhibition of p53-mediated transactivation and cell cycle arrest by E1A through its p300/CBP-interacting region. *Oncogene.* 1997;14(9):1047-57.

10. Banu SK, Starzinski-Powitz A, Speights VO, Burghardt RC, and Arosh JA. Induction of peritoneal endometriosis in nude mice with use of human immortalized endometriosis epithelial and stromal cells: a potential experimental tool to study molecular pathogenesis of endometriosis in humans. *Fertil Steril.* 2009;91(5 Suppl):2199-209.

11. Arosh JA, Lee J, Balasubbramanian D, Stanley JA, Long CR, Meagher MW, et al. Molecular and preclinical basis to inhibit PGE2 receptors EP2 and EP4 as a novel nonsteroidal therapy for endometriosis. *Proc Natl Acad Sci U S A.* 2015;112(31):9716-21.

12. Gou Y, Li X, Li P, Zhang H, Xu T, Wang H, et al. Estrogen receptor beta upregulates CCL2 via NF-kappaB signaling in endometriotic stromal cells and recruits macrophages to promote the pathogenesis of endometriosis. *Hum Reprod.* 2019;34(4):646-58.

13. Zhang H, Li L, Chen Q, Li M, Feng J, Sun Y, et al. PGC1beta regulates multiple myeloma tumor growth through LDHA-mediated glycolytic metabolism. *Mol Oncol.* 2018;12(9):1579-95.

14. Chen P, Mamillapalli R, Habata S, and Taylor HS. Endometriosis stromal cells induce bone marrow mesenchymal stem cell differentiation and PD-1 expression through paracrine signaling. *Mol Cell Biochem.* 2021;476(4):1717-27.

15. Ramezani G, Norouzi A, Arabshahi SKS, Sohrabi Z, Zazoli AZ, Saravani S, et al. Study of medical students' learning approaches and their association with academic performance and problem-solving styles. *J Educ Health Promot.* 2022;11:252.

**Table S1. Sequences of primers for the real time quantitative PCR (qPCR)**

| Gene | Species | Analysis | Forward primer (5'→3') | Reverse primer (5'→3') |
| --- | --- | --- | --- | --- |
| β-actin | Human | mRNA | gatgcagaaggagatcactgc | atactcctgcttgctgatcca |
| ERα | Human | mRNA | gggaagctactgtttgctcct | ttgaggcacacaaactcctct |
| ERβ | Human | mRNA | atgatgatgtccctgaccaag | acatcagccccatcattaaca |
| EZH2 | Human | mRNA | gatgatggagacgatcctgaa | cttctgctgtgcccttatctg |
| IL1β | Human | mRNA | tgggataacgaggcttatgtg | gaacaccacttgttgctccat |
| IL6 | Human | mRNA | tccaaagatggctgaaaaaga | gctctggcttgttcctcacta |
| TNFα | Human | mRNA | tagcccatgttgtagcaaacc | aggacctgggagtagatgagg |
| PGE2 | Human | mRNA | catcagttgagcactgcaaga | tctggcaaaactttcgaagaa |
| β-actin | Human | ChIP | tgtagcctgtacatcctccca | ggtctgcagtttgtacctgga |
| ERα | Human | ChIP | atccgtctttcgcgtttattt | aaagagcagcttccctgaact |
| TNFα | Human | ChIP | ctccagggtcctacacacaaa | tctgtctcggtttcttctcca |
| β-actin | Mouse | mRNA | tcttgggtatggaatcctgtg | atctccttctgcatcctgtca |
| ERα | Mouse | mRNA | atgtgctatggccaacttctg | caagcttcctcttcagggtct |
| ERβ | Mouse | mRNA | atgtgctatggccaacttctg | caagcttcctcttcagggtct |
| EZH2 | Mouse | mRNA | atcaggatggcactttcattg | tctccatcatcgtcatcatca |
| TNFα | Mouse | mRNA | ccaccacgctcttctgtctac | gctacaggcttgtcactcgaa |

FIGURE S1

**Figure S1. Representative pictures of full blots for Western Blotting.** (a) Full blots for Figure 1c. (b) Full blots for Figure 2g. (c) Full blots for Figure 3c. (d) Full blots for Figure 6d.

FIGURE S2

**Figure S2. EZH2 regulates TNFα expression through estrogen receptors in HEEC cells.** (a) Immortalized human HEEC cells were infected by either control (CTL), or knockdown lentivirus for EZH2 (shEZH2) for 48 hours, followed by analysis of mRNA levels by qPCR. (b) Immortalized human HESC cells were infected by either control (CTL), or knockdown lentivirus for ERα (shERα) or ERβ (shERβ) for 48 hours, followed by analysis of mRNA levels by qPCR. n=4. *, *P*<0.05 vs. CTL group.

FIGURE S3

**Figure S3.** **EZH2 inhibitor GSK343 partly suppresses pro-inflammatory cytokine release, mimicking the effects gene knockdown of ERβ and EZH2.** HESC cells were treated with control (CTL), knockdown lentivirus for either ERβ (shERβ) or EZH2 (shEZH2), or 5μM of EZH2 inhibitor GSK343 for 2 days, and then harvested for biological assay. (a) mRNA levels by qPCR, n=4. (b-e) secretion of pro-inflammatory cytokines for IL1β (b), IL6 (c), TNFα (d) and PGE2 (e), n=5. *, *P*<0.05 vs. CTL treatment.
